# Effects of the combination of traditional Thai massage, scapular stabilization exercise, and chest mobilization in subjects with forward head posture: a single-blinded randomized clinical trial

**DOI:** 10.1186/s12998-023-00506-z

**Published:** 2023-08-21

**Authors:** Vitsarut Buttagat, Sujittra Kluayhomthong, Pattanasin Areeudomwong

**Affiliations:** Department of Physical Therapy, School of Integrative Medicine, Mae Fah Luang University, 333 Moo1, Tasud Sub-district, Muang District, Chiang Rai, 57100 Thailand

**Keywords:** Thai massage, Scapular stabilization exercise, Chest mobilization, Forward head posture

## Abstract

**Background:**

Forward head posture (FHP) is  a common condition where the head appears to be positioned in front of the vertical midline of the body. FHP is associated with shortening of the neck extensors and pectoral muscles, and the deep neck flexors and shoulder retractors are weakened. FHP is also found to cause decreases in respiratory function. Few clinical trials have investigated the effects of combination treatments to alleviate these problems. The aim of this study was to examine the effects of combination of traditional Thai massage, scapular stabilization exercise, and chest mobilization on forward head angle (FHA), forced vital capacity (FVC), and cervical flexion in subjects with FHP.

**Methods:**

Forty-eight subjects with FHP were randomly allocated to a treatment group receiving a Combination of Traditional Thai massage, Scapular stabilization exercise, and Chest mobilization (CTSC group) (n = 24) and a control group (relaxed by lying supine) (n = 24). FHA, FVC, and cervical flexion were measured before and after the four-week intervention (Week 4) and one month after the intervention period (Week 8).

**Results:**

The CTSC group showed statistically significantly greater improvement in FHA and cervical flexion than the control group at Week 4 (FHA, mean difference − 6.05; 95% CI − 8.03, − 4.07; cervical flexion, mean difference 6.84; 95% CI 3.14, 10.55) and Week 8 (FHA, mean difference − 4.64; 95% CI ( − 6.71, − 2.58); cervical flexion, mean difference 5.21; 95% CI 0.84, 9.58). There were no significant between-group differences in FVC at week 4 (mean difference 0.09; 95% CI − 0.06, 0.23) and week 8 (mean difference 0.04; 95% CI − 0.11, 0.19).

**Conclusion:**

This study showed that CTSC had a positive effect on FHA and cervical flexion in subjects with FHP.

*Trial registration:* Thai Clinical Trials Registry (TCTR) (Identification number: TCTR20211119001), registered 19 November 2021; https://www.thaiclinicaltrials.org/show/TCTR20211119001.

**Supplementary Information:**

The online version contains supplementary material available at 10.1186/s12998-023-00506-z.

## Background

Forward head posture (FHP) is a common condition where the head appears to be positioned in front of the vertical midline of the body. It causes an increased load on the cervical spine, which can lead to spinal degeneration [1]. This condition is associated with frequent use of smartphones and computers for long periods of time [1–3]. A previous study of the incidence of FHP found that ranged from 66 to 85% in healthy participants [4]. Joshi and Sheth [3] found FHP in approximately 63% of Indian high school students. In addition, the prevalence of FHP among physical therapy students at Mae Fah Luang University, was found to be 65.31% [5]. Previous studies have reported that individuals with FHP present with shortening of the neck extensors, sternocleidomastoid, upper trapezius, and pectoral muscles as well as weakness in the deep neck flexors, rhomboids, serratus anterior, middle trapezius, and lower trapezius muscles [1–4, 6–9]. Moreover, FHP has been reported to be associated with rib cage elevation resulting from sternocleidomastoid muscle overactivity, the reduction of lower rib movement during respiration, decreased thoraco-abdominal mobility and chest expansion, decreased length of the intercostal muscles, and respiratory weakening, which can lead to impairments of the respiratory muscles [6–9]. Koseki et al. [8] and Han et al. [10] found that subjects with FHP had significantly lower forced vital capacity (FVC) than subjects with normal head posture.

Given the main problems of FHP outlined above, a combination of treatment techniques is essential to effectively remedy these issues. Kendall et al. [11] suggested that in order to correct postural alignment, e.g., FHP, the therapeutic techniques that have been advocated for the strengthening of the weak postural muscles and the relaxing or stretching of shortened muscles. Additionally, to improve respiratory function, breathing retraining exercises were recommended [12]. The current conservative treatments of FHP include postural correction, scapular stabilization exercise (SSE), stretching exercises, and massage [11].

SSE is comprised of a set of exercises used to restore the position, motor control, and movement pattern of the scapula to improve scapular stability [13, 14]. Kang et al. [2] revealed that SSE can be considered and used as an effective intervention to improve the postural and muscular imbalance in subjects with FHP. Traditional Thai massage (TTM) is the most common type of massage in Thailand, which uses gentle acupressure and stretches to relax the muscles [15, 16]. This massage technique has the biomechanical benefit of addressing trigger points in the body, along with passive muscle elongation [15–17]. TTM is performed with the fully clothed patient on a mat [16, 18]. The therapist generally uses the palms or the thumbs to press the muscles along the patient’s body’s ten hypothesized major energy channels (Sen Sib) with a combination of stretching at the end of the session [15, 16, 18]. Chaithavuthi and Muangsiri [19] reported that TTM can relieve muscle tension and pain, increase joint mobility and flexibility, and reduce stiffness. Plakornkul et al. [20] revealed that TTM were associated with significant increases in blood flow and skin temperature. Buttagat et al. [21] found that TTM can significantly decrease electromyographic activity (EMG) and the feeling of muscle tension in participants with upper back pain associated with myofascial trigger points (MTrPs). Chest mobilization (CM) is a form of exercise designed to increase chest wall mobility and thoracic cage expansion and improve ventilation by moving the upper limbs and body in combination with deep breathing [22–24]. Sreejith and Praveena [25] found that, in elderly population, CM was associated with significant improvement in pulmonary function after treatment sessions.

Of the several studies of the therapeutic techniques used for FHP, few studies have evaluated the effects of a combination of treatment techniques for individuals with FHP. Thus far, no studies have yet addressed the effects of the Combination of Traditional Thai massage, Scapular stabilization exercise, and Chest mobilization (CTSC), which comprise a group of interventions that can address all the main problems of subjects with FHP. Therefore, this study was performed to investigate the effects of the combination of TTM, SSE, and CM on FHA, FVC, and cervical range of motion (CROM) in participants with FHP.

## Methods

### Study design

This study was designed as a randomized controlled trial between December 2021 and September 2022 in the Institute of Thai-Chinese Traditional Medicine, Mae Fah Luang University Hospital, Mae Fah Luang University, Thailand. This trial was approved by the Ethics Committee for Human Research at Mae Fah Luang University. The present study was prospectively registered (TCTR20211119001).

### Setting

This study was conducted in the the Institute of Thai-Chinese Traditional Medicine, Mae Fah Luang University hospital, Mae Fah Luang University, Thailand.

### Participants

Participants with FHP were recruited from Chiang Rai province, through poster advertisement. Inclusion criteria included participants aged between 18 and 60 years with FHP (forward head angle [FHA] ≥ 46) who were willing to cooperate with the study. FHA measured from the vertical line to a line connecting the tragus and the seventh cervical vertebra. Participants with FHA greater than or equal to 46 degrees were arranged to FHP [26]. Participants were excluded if they had anatomy deformity or abnormal alignment (an abnormal, asymmetrical head or neck position), such as cervical spondylolisthesis or torticollis, determined by case history acquired through interview and physical examination or examining the medical records from the participant's primary healthcare hospital, neck pain, a history of cervical radiculopathy, a history of neurological disorders, and other conditions that could be contraindicated for TTM, such as contagious skin diseases. Written informed consent was obtained from all participants.

### Sample size calculation

The calculation of sample size was based on our pilot study, which compared the effects of the combination of TTM, SSE, and CM (n = 4) with that of a control group (n = 4) for participants with FHP. A standard FHA deviation of 3.92 degrees for the CTSC group and of 2.6 degrees for the control group was observed. These variances were used to estimate the sample size needed to detect a 3.44-degree change (between the intervention and control group) in FHA. Considering a dropout rate of 20%, it was estimated that 24 participants per group were needed to obtain 90% power with a 5% significance level.

### Randomization

Participants were randomly allocated to receive the combination treatment of TTM, SSE, and CM (CTSC group) or to the control treatment (rest on bed) using block randomization (1:1 ratio) with block sizes of 2, 4, and 6. Groups were assigned using a pre-generated random assignment scheme enclosed in envelopes. Randomization was conducted by a research assistant who was unaware of the intervention and outcome assessment processes.

### Interventions

**Fig. 1:**
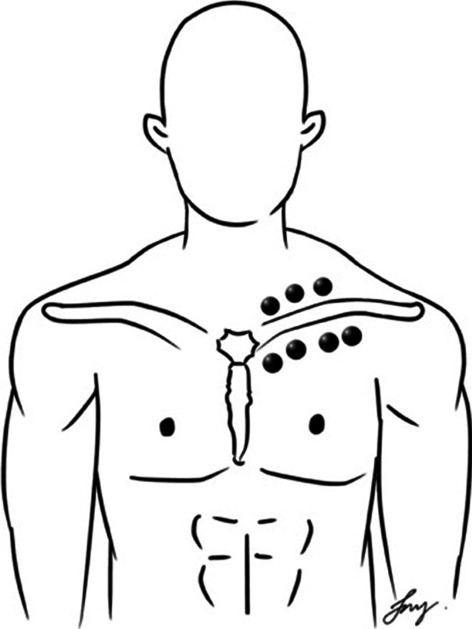
TTM points used in the present study (The anterior side of the upper trunk)

**Fig. 2:**
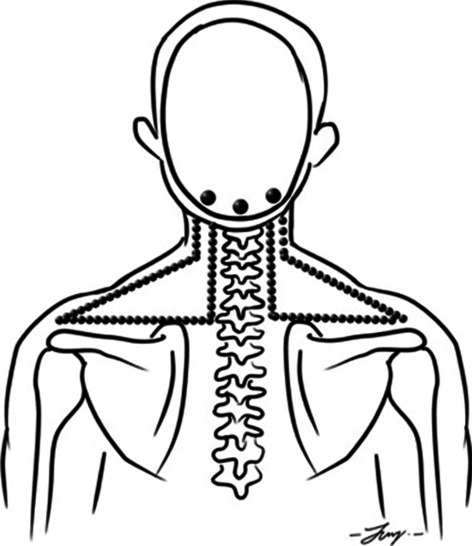
TTM points used in the present study (The posterior side of the upper trunk)

**Fig. 3:**
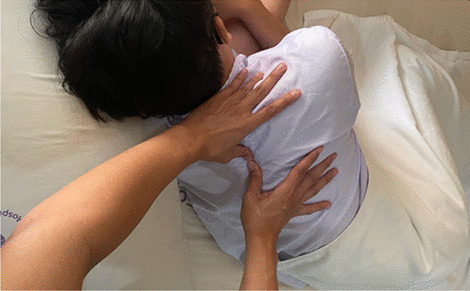
The massage therapist used the thumb to apply pressure for 5 s per point

**Fig. 4:**
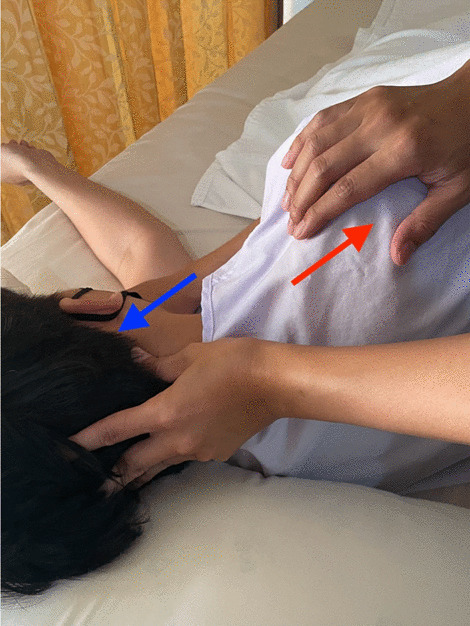
The massage therapist used the thumb to apply pressure, along with passive muscle elongation

**Fig. 5:**
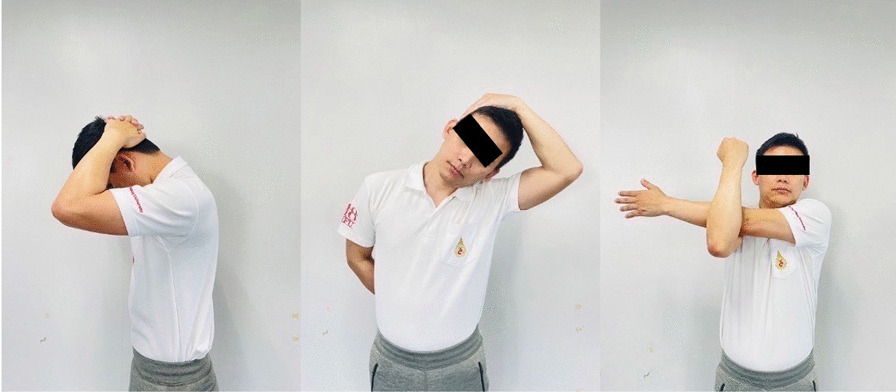
Stretching exercises for upper trapezius, posterior neck, and rhomboids

**Fig. 6:**
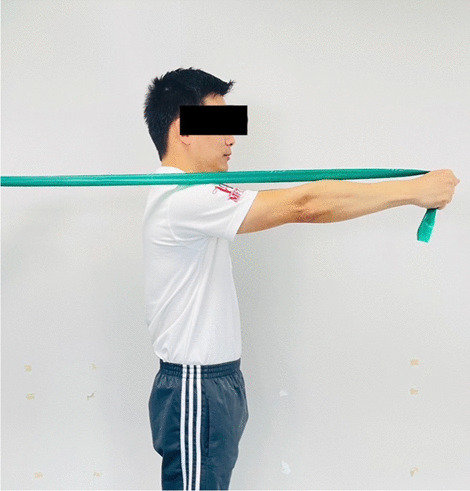
Scapular protraction with an elastic band

**Fig. 7:**
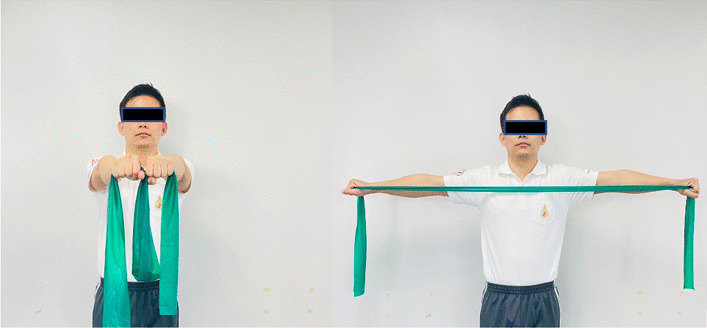
Scapular retraction with an elastic band

**Fig. 8:**
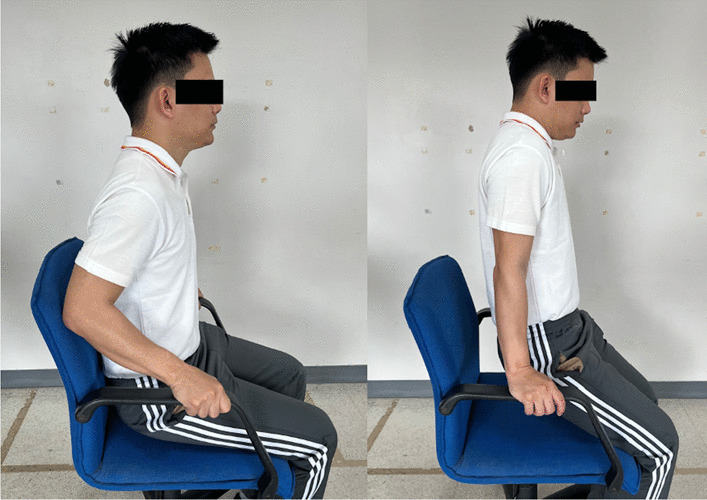
Scapular depression

**Fig. 9:**
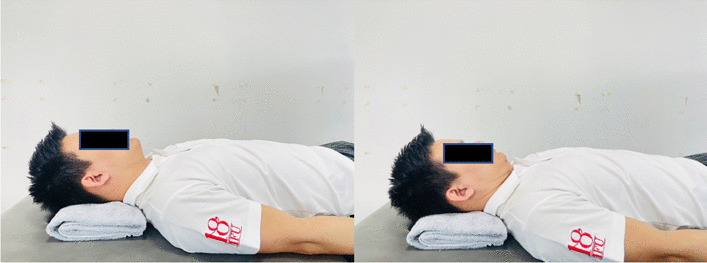
Supine chin tucking

**Fig. 10:**
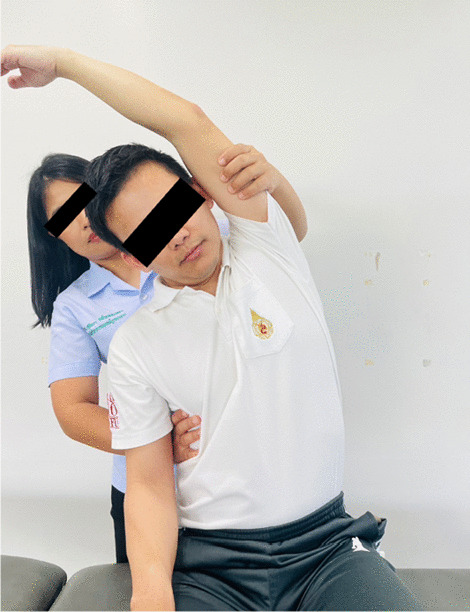
Passive lateral costal chest wall mobilization

**Fig. 11:**
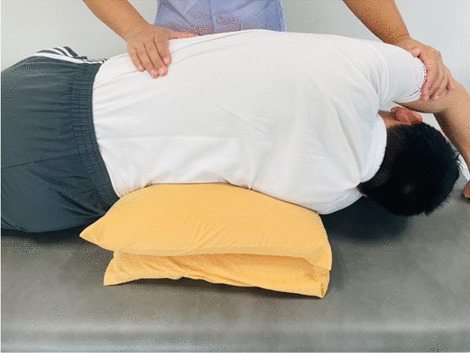
Passive lateral costal chest wall mobilization in the side-lying position

**Fig. 12:**
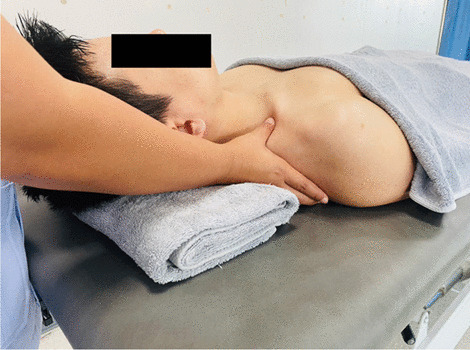
First rib mobilization

In the CTSC group, the intervention consisted of the following three treatment techniques: TTM, SSE, and CM. These treatment techniques were provided for 12 sessions over a period of four weeks. TTM was administered 15 min per session to the upper trunk area by a trained massage therapist. TTM points used in the present study were located along five lines on each side of the upper trunk (Figs. 1 and 2). The massage therapist used the thumb or palm to apply pressure for 5 s per point until the participants begin to feel slight discomfort or pain (Fig. 3), along with passive muscle elongation by positioning (Fig. 4) in order to address the trigger points or muscle tightness in the upper body. This sequence can be repeated several times for each massage point [27]. The SSE program was performed for 15 min per session, consisting of stretching exercises and scapular exercises. Participants performed three sets of five repetitions of stretching exercises of the upper trapezius, rhomboids, and posterior neck, maintaining each position for 20 s (Fig. 5). The scapular exercises included three sets of 10 repetitions of scapular protraction with an elastic band, scapular retraction with an elastic band, scapular depression, and supine chin tucking. For the scapular protraction with an elastic band, the elastic band was anchored at the shoulder level. Participants pushed the arm forward as far as possible and then held this position for 5 s (Fig. 6). For the scapular retraction with an elastic band, the participants held both ends of the elastic band with the elbows straight while pulling the arms apart and toward the side, holding this position for 5 s (Fig. 7). The method used to determine the suitable resistance level of the elastic band for the scapular protraction and retraction exercises for each participant have been previously described by Hibberd et al. [28]. For scapular depression, the participants sat in an armchair with the feet flat on the floor, arms pushed down on the armrests, and lifted the body up as far as possible; participants then held this position for 5 s (Fig. 8). For supine chin tucking, the participants were in the supine position with a rolled-up towel under the neck at the base of the skull in order to keep the resting head in a neutral position and then nodded the chin toward the chest and held this position for 5 s (Fig. 9). The CM program included three sets of six repetitions of passive lateral costal chest wall mobilization (both left and right side), passive lateral costal chest wall mobilization in the side-lying position (both left and right side), and first rib mobilization. For passive lateral costal chest wall mobilization, while the participants were sitting with one arm raised overhead, the therapist passively bent the participants’ trunk and the raised arm to the opposite side during a deep inhalation; this was then repeated on the other side (Fig. 10). For passive lateral costal chest wall mobilization in the side-lying position, while the participants were in this position on the pillows, the therapist passively brought the rib cage to the opposite side, bending the proximal humerus during a deep inhalation; this was then repeated on the other side (Fig. 11). For first rib mobilization, the therapist pressed the left and right first ribs downward and medially during deep exhalation (Fig. 12).

In the control group, the participants relaxed by lying supine in the same environment and for the same period as the CTSC group. In addition, all participants in both groups received a pamphlet describing self-care for people with FHP (Additional file 1). However, they were not advised to perform any exercises at home. Additionally, the participants were asked to inform if they had done the exercise or other treatment at home.

### Outcome measures

All outcome measures were carried out by a research assistant who was blinded to group allocation. The measurements were taken at baseline at the end of the intervention period and one month after the end of the intervention period (one month follow-up).

The primary outcome was FHA, measured from the vertical line to a line connecting the tragus and the seventh cervical vertebra [26]. The sagittal plane of the upper body of each participant was captured using a digital camera. Markers were placed over the tragus of the right ear and the spinous process of the C7. The FHA measurement procedure used in the present study has been described in more detail by Thigpen et al. [26].

The secondary outcomes included cervical flexion range of motion (CROM), and FVC. Cervical flexion was measured using the CROM instrument. The CROM instrument was placed on the participant’s head while they were in the sitting position. The participant was instructed to move the neck slowly into flexion until they had reached maximum flexion. The validity and reliability of the data obtained with the CROM instrument was shown to be satisfactory [29] Cervical flexion measurement was repeated three times, and the resulting values were averaged for analysis.

FVC was measured by using a spirometer (Vyaire medical, Germany) based on the American Thoracic Society’s recommendation for diagnostic spirometry. While seated on a chair, the participants were instructed to inhale as deeply as possible through the mouthpiece and then exhale as forcefully as possible. This procedure was repeated three times, and the average value was used for analysis.

### Statistical analysis

Statistical analyses were performed using the SPSS 20.0 software package (version 20.0; IBM, New York, US). The Shapiro–Wilk test was used to verify the distribution of data. Descriptive statistics were expressed as mean ± standard deviation. A repeated measures ANOVA was used to verify the differences within each group. Between-group differences were assessed using analysis of covariance (ANCOVA) with the baseline score included as the covariate. The statistical significance was set at *p *< 0.05 for all tests.

## Results

**Fig. 13:**
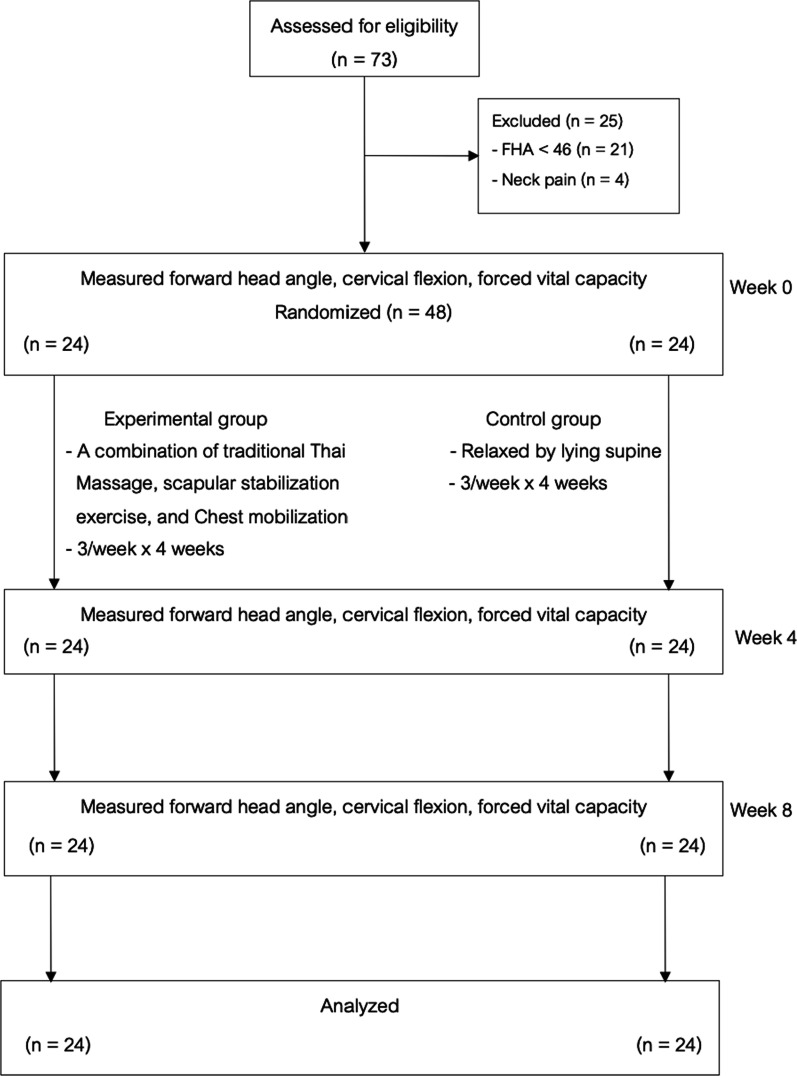
The CONSORT flow diagram

As shown in the CONSORT flow diagram (Fig. 13), a total of 73 participants responded to the advertisements and were screened for eligibility. Forty-eight individuals were included and randomized (24 participants to the CTSC group and 24 participants to the control group) (Table 1).

**Table 1:** The baseline data of participant demographics and characteristics

Characteristic	Randomised (n = 48)	
	CTSC (n = 24)	Control (n = 24)
Age (years), mean (SD)	24.83 (5.76)	24.67 (5.98)
Gender; number of female (%)	15 (62.5)	16 (66.67)
Weight (kg), mean (SD)	67.08 (11.46)	66.56 (14.50)
Height (cm), mean (SD)	163.75 (13.46)	163.71 (8.75)

*CTSC* Combination of traditional Thai massage, scapular stabilization exercise and chest mobilization

### Participant compliance with trial interventions and data collection protocols

Dropouts were defined as participants who did not attend the final study visit. In the present study, all participants allocated to the CTSC group attended all sessions of the CTSC and no participants in the control group withdrew from the current study. Therefore, the compliance rate was 100%. All data were collected as intended and all participants were included in the analyses.

### Effects of intervention

The FHA, and cervical flexion all showed significant improvements with treatment among participants in the CTSC group after the end of the treatment period (Week 4) and at one month after the end of the treatment period (Week 8) (*p *< 0.05). In contrast, there was no evidence for a within-group difference in changes in FVC values in the CTSC group at Week 4 and Week 8 (*p *> 0.05). For the control group, no statistically significant difference in changes in terms of all parameters were observed **(**Table 2). A comparison of the adjusted post-test values of each assessment time point (Week 4 and Week 8) for FHA, muscle tension, and cervical flexion between the CTSC and control groups indicated a significantly greater improvement in these parameters in the treatment group (*p *< 0.05). There was no statistically significant difference in FVC between the study groups (Table 3).

**Table 2:** Comparison of the outcome measures between baseline (pre-test) and post-test assessments in the CTSC and control groups (Repeated measures ANOVA)

Outcome measures	Groups	Baseline	Week 4 (Post-test 1)	Week 8 (Post-test 2)
Forward head angle (FHA): Mean ± SD	CTSC	49.03 ± 2.82	41.57 ± 4.07*	42.73 ± 4.21*
	Control	47.98 ± 2.17	47.56 ± 2.28	47.40 ± 2.45
Forced vital capacity (FVC): Mean ± SD	CTSC	3.41 ± 0.68	3.49 ± 0.66	3.44 ± 0.69
	Control	3.36 ± 0.78	3.36 ± 0.71	3.36 ± 0.72
Cervical range of motion (CROM): Mean ± SD	CTSC	53.20 ± 11.64	61.62 ± 9.87*	62.04 ± 10.35*
	Control	57.15 ± 11.13	57.40 ± 9.71	58.89 ± 8.39

*CTSC* Combination of Traditional Thai massage, Scapular stabilization exercise and Chest mobilization

*Significant improvement from baseline levels (*P *< 0.05)

**Table 3:** Comparison of mean post-test measures at week 4 and week 8 between the CTSC and control groups after adjustment for differences in baseline values (ANCOVA)

Outcome	Week 4 (Post-test 1)			Week 8 (Post-test 2)		
	CTSC	Control	Difference (95% CI)	CTSC	Control	Difference (95% CI)
Forward head angle (FHA)	41.54	47.59	− 6.05* ( − 8.03, − 4.07)	42.75	47.39	− 4.64* ( − 6.71, − 2.58)
Forced vital capacity (FVC)	3.47	3.38	0.09 ( − 0.06, 0.23)	3.42	3.38	0.04 ( − 0.11, 0.19)
Cervical range of motion (CROM)	62.94	56.09	6.84* (3.14, 10.55)	63.07	57.86	5.21* (0.84, 9.58)

*CTSC* Combination of traditional Thai massage, Scapular stabilization exercise and Chest mobilization

*Significant difference between groups (*P *< 0.05)

## Discussion

This is the first study to evaluate the effectiveness of a four-week rehabilitation program including TTM, SSE, and CM on FHA, FVC, and cervical flexion among individuals with FHP. Within group analysis found that in the CTSC group, remarkable and statistically significant improvements were seen in FHA and cervical flexion after receiving the treatment program. These improvements were maintained for one month following the treatment program. At Week 4 and Week 8, FVC had not significantly improved. When comparing between the groups, significant differences in FHA and cervical flexion were observed at Week 4 and Week 8. These findings extend the recommendations of Ruivo et al. [30] and Lynch et al. [31], who suggested the following as an effective method to improve FHP: stretching or releasing the shortened neck extensors, pectoralis, upper trapezius, and levator scapulae muscles; and strengthening the scapula stabilizers and deep cervical flexor muscles.

These results are compatible with those of Harman et al. [32], who investigated the effects of a 10-week targeted home exercise program consisting of strengthening the deep cervical flexors and shoulder retractors as well as stretching the cervical extensors and pectoral muscles to improve FHP and neck flexion range of motion in normal adults. They found that such a targeted home exercise program could improve both FHP and neck flexion in this population. Ruivo et al. [30] found that after participants underwent a 32-week stretching and SSE program, FHP improved. Fathollahnejad et al. [33] stated that a six-week combination of manipulation and SSE could improve pain, function, and head posture (FHA) in patients with forward head and rounded shoulder postures (FHRSP). Im et al. [34] showed that a four-week SSE program improved FHP, upper trapezius muscle activity, serratus anterior muscle activity, neck disability, pain intensity, and quality of life in individuals with neck pain and FHP. Kang et al. [2] reported that SSE and neck stabilization exercises could have positive effects on FHP and muscle activation (decreasing muscle activation on the upper trapezius and increasing muscle activation on the lower trapezius and serratus anterior). Kim et al. [35] revealed that an elastic band exercise program improved FHP and the length of the pectoralis major muscle in participants with FHRSP.

The improvements in FHA and cervical flexion in the current study may be due to the treatment program provided in this study, perhaps correcting the patterns of muscle imbalances between opposing muscle groups caused by FHP [30]. In the present study, TTM was chosen to release and lengthen the shortened muscles, e.g., neck extensors, pectoralis, upper trapezius, and levator scapulae muscles [21, 36, 37], and SSE was used to strengthen the weakened muscles, e.g., rhomboids, serratus anterior, lower and middle trapezius, and deep cervical flexor muscles [30, 32, 35], consequently correcting the muscle imbalances, and improving FHP and cervical flexion. Additionally, CM was also included in our treatment program in order to increase the length of the intercostal muscles, improve the flexibility and mobility of the chest wall, and correct the misalignment of the thoracic spine and rib cage [38, 39]. Furthermore, the decrease in FHA observed in this study may be related to the change in the serratus anterior and upper trapezius muscle activity. Im et al. [34] showed that SSE can enhance serratus anterior muscle activity and reduce upper trapezius muscle activity in patients with neck pain associated with FHP, and Holtermann et al. [40] found that such changes lead to functional changes associated with head and neck posture.

Unexpectedly, a significant change in FVC post-treatment (Week 4) and at follow-up (Week 8) was not observed. This finding is inconsistent with those of previous studies. Pawaria et al. [6, 7] reported that a six-week cervical stabilization exercise program was effective in improving FHP, pulmonary function, and respiratory muscle strength in patients with chronic neck pain associated with FHP. Joshi and Sheth [3] demonstrated that 12 weeks of McKenzie self-treatment could improve FHP and increase peak expiratory flow rate (PEFR) in adolescent girls with FHP. These conflicting findings may be due to the duration of treatment provided in the present trial (a four-week program) not being sufficient to make changes in FVC in subjects with FHP. The other possibility is that the sample demographic in this study was asymptomatic. Therefore, such findings of the current study may not be in line with those of previous studies [3, 6, 7].

Some limitations in this study should be acknowledged. First, the treatment program was conducted for only four weeks, which might be inadequate to draw a conclusion regarding the changes in FVC after participants received CTSC. In future studies, this treatment program should be provided for longer periods. Second, it is difficult to generalize the findings of the current study given that it involved only young participants. The treatment effects found in this study might not extend to an older population. Further studies should involve participants of different ages. Finally, although a research assistant who assessed all outcomes was unaware of the assigned groups of the participants, blinding of the participants was not possible.

## Conclusion

Based on the results of the current study, four weeks of CTSC was shown to reduce FHA as well as increase cervical flexion range of motion in participants with FHP. This intervention also provided positive short-term effects in terms of these parameters. However, FVC was not affected by CTSC. It is believed that the findings of the present study will provide insight for physical therapists and other healthcare providers for FHP management. Given these promising findings, this rehabilitation program may therefore be considered for use as an alternative method in treating FHP.

### Supplementary Information

**Additional file 1.** Pamphlet describing self-care for people with FHP.

## Data Availability

The dataset used in this study are available from the corresponding author on reasonable request.

## Abbreviations

FHP: Forward head posture
TTM: Traditional Thai massage
SSE: Scapular stabilization exercise
CM: Chest mobilization
FHA: Forward head angle
FVC: Forced vital capacity
CTSC: A combination of TTM, SSE, and CM
NDI: Neck disability index
CROM: Cervical range of motion
ANOVA: Analysis of variance
ANCOVA: Analysis of covariance
