# Supplementary material for: Effects of the combination of traditional Thai massage, scapular stabilization exercise, and chest mobilization in subjects with forward head posture: a single-blinded randomized clinical trial
Source: Chiropr Man Therap. 2023 Aug 21;31:31. doi: 10.1186/s12998-023-00506-z (PMC10441760; doi:10.1186/s12998-023-00506-z)
Supplement: Supplementary file 1 — Additional file 1. Pamphlet describing self-care for people with FHP. [file 12998_2023_506_MOESM1_ESM.docx]

| \| 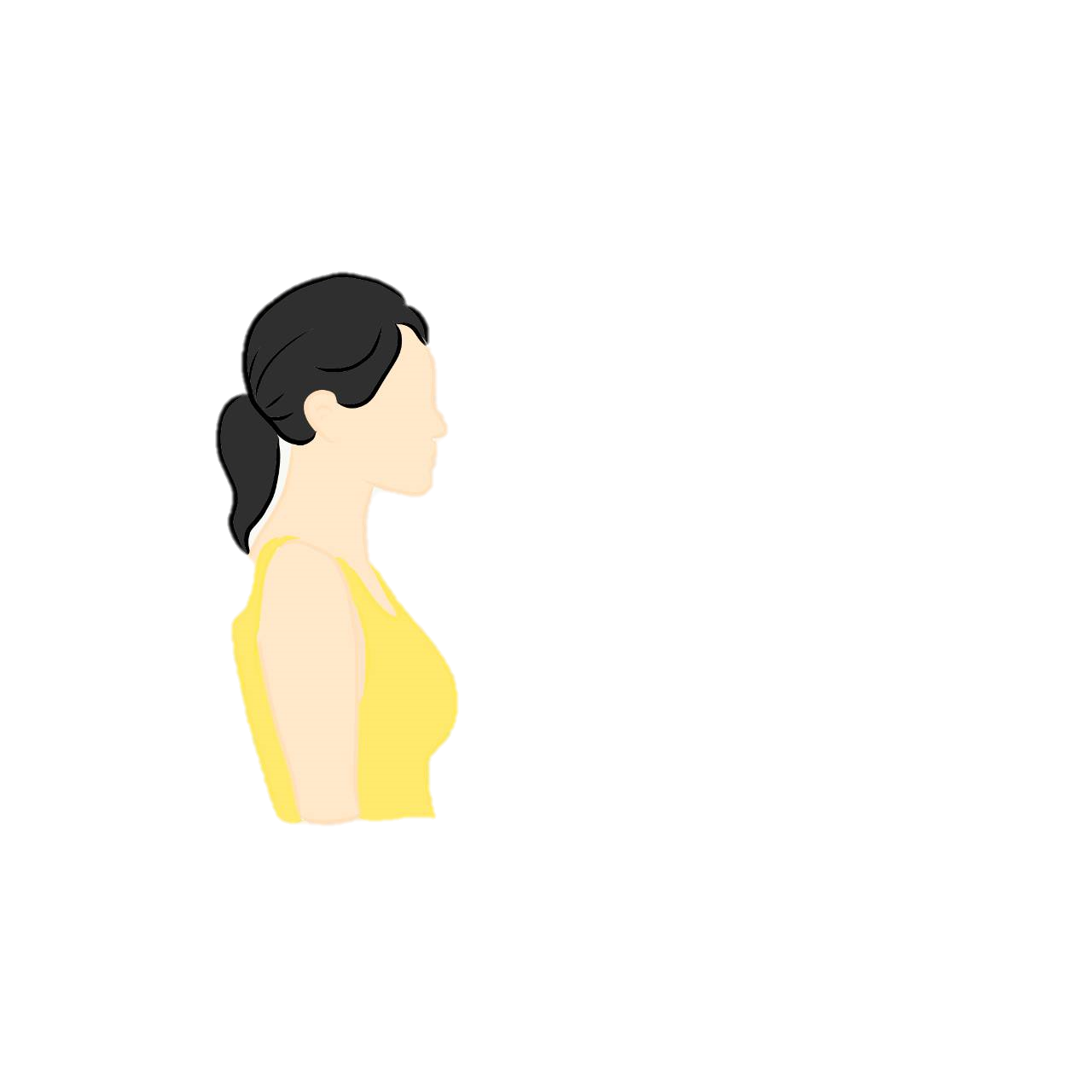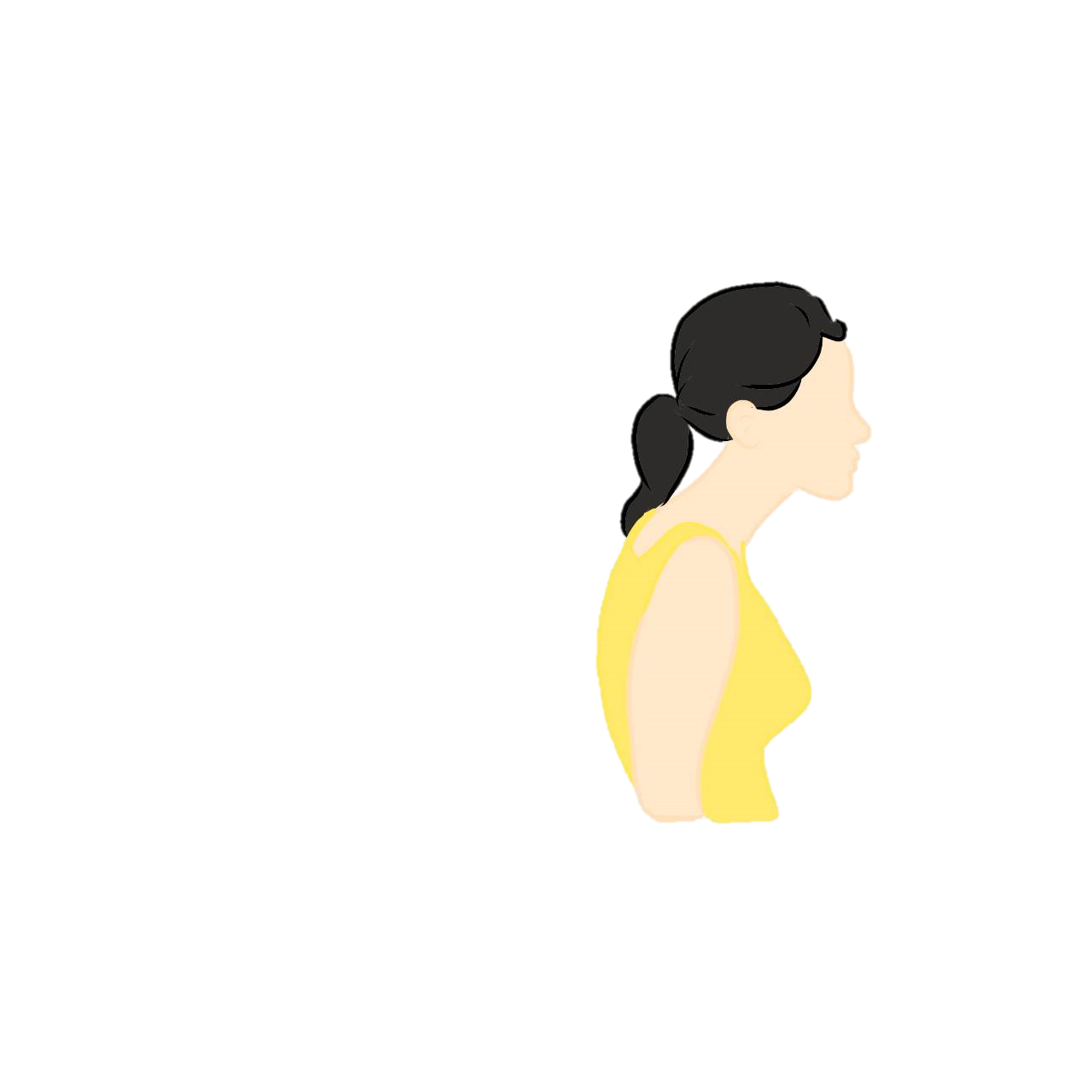  **FHP is a common condition where the head appears to be positioned in front of the vertical midline of the body.**  **This condition is associated with frequent use of smart phones and computers for long periods of time.**  **Forward head posture (FHP)** \| FHP can lead to…. 1. Neck pain or discomfort  2. Spinal degeneration  3. can result in shoulder pain and dysfunction  3. Impairments of the respiratory muscles \|  \| \| --- \| --- \| --- \| | 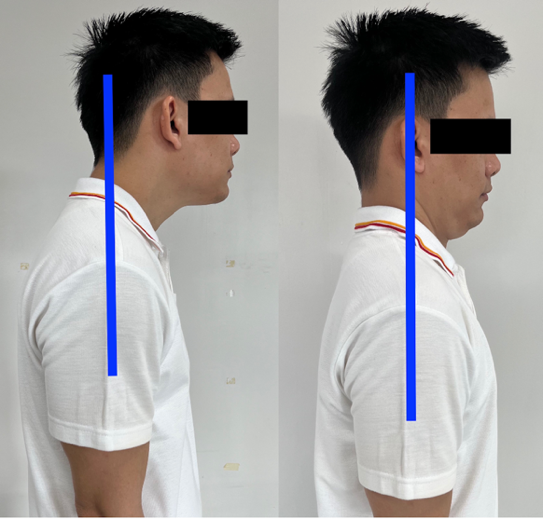  When you are sitting or standing, your ear canal (external auditory meatus) must be aligned with the midline of your body and the gaze of your eyes must be forward.  **How to Fix FHP** | 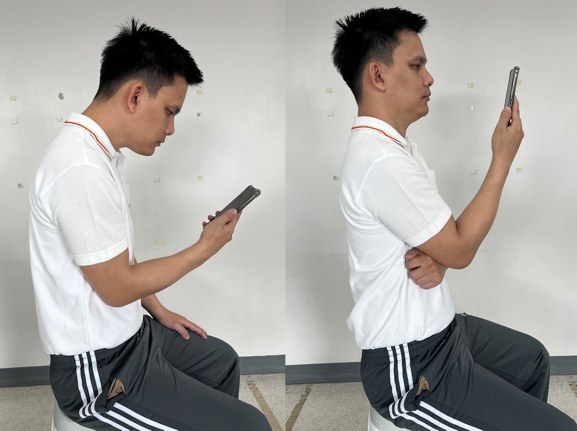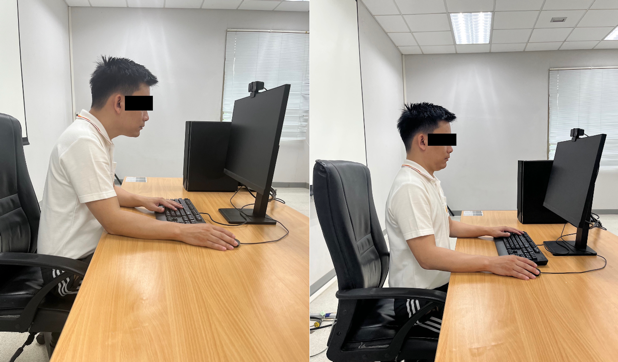 Move the cellphone up until your cellphone is right in front of your eyes so the head does not have to be tilted forward.  - Head upright and over your shoulder without straining forward or backward.  - Place the computer monitor directly in front of you, about an arm's length from screen.  - Top of screen should be at or slightly below eye level. |
| --- | --- | --- | --- | --- | --- |
